# Association of HDAC8 Expression with Pathological Findings in Triple Negative and Non-Triple Negative Breast Cancer: Implications for Diagnosis

**DOI:** 10.29252/ibj.24.5.283

**Published:** 2020-05-02

**Authors:** Mohammad-Nazir Menbari, Karim Rahimi, Abbas Ahmadi, Samira Mohammadi-Yeganeh, Anvar Elyasi, Nikoo Darvishi, Vahedeh Hosseini, Mohammad Abdi

**Affiliations:** 1 Cellular and Molecular Research Center, Research Institute for Health Development, Kurdistan University of Medical Sciences, Sanandaj, Iran;; 2 Department of Molecular Biology and Genetics, Gene Expression and Gene Medicine, Aarhus University, Aarhus, Denmark;; 3 Interdisciplinary Nanoscience Center, Aarhus University, Aarhus, Denmark;; 4 Medical Nanotechnology Research Center, Shahid Beheshti University of Medical Sciences, Tehran, Iran;; 5 Department of Biotechnology, School of Advanced Technologies in Medicine, Shahid Beheshti University of Medical Sciences, Tehran, Iran;; 6 Department of Surgery, Faculty of Medicine, Kurdistan University of Medical Sciences, Sanandaj, Iran;; 7 Department of Clinical Biochemistry, Faculty of Medicine, Kurdistan University of Medical Sciences, Sanandaj, Iran

**Keywords:** Breast cancer, HDAC8, Triple negative breast cancer

## Abstract

**Background:**

Previous data have shown the tumorigenicity roles of HDAC8 in breast cancer. More recently, the oncogenic effects of this molecule have been revealed in TNBC. The present study aimed to determine the diagnostic value of HDAC8 for the differentiation of TNBC from nTNBC tumors.

**Methods:**

A total of 50 cancerous and normal adjacent tumor specimens were obtained, and the clinical and pathological findings of studied subjects were recorded. The expression of *HDAC8* gene was determined by qRT-PCR. Also, immunohistochemical staining was performed on tissue samples.

**Results:**

Our results showed that the expression of* HDAC8* in breast cancer tissues was significantly higher than the normal adjacent tissues (*p* = 0.0011). *HDAC8* expression was also observed to be higher in TNBC patients than nTNBC group (*p* = 0.0013). In addition, in the TNBC group, there was a significant association between the *HDAC8* overexpression and tumor characteristics, including tumor size (*p* = 0.039), lymphatic invasion (*p* = 0.01), tumor grade (*p* = 0.02), and perineural invasion (*p* < 0.05). The cut-off value was fixed at 0.6279 r.u., and the corresponding sensitivity and specificity were found to be 73.91% and 70.37%, respectively.

**Conclusion:**

According to the findings, among the other markers, HDAC8 oncogene may be used as a potential tumor marker in diagnosis of TNBC tumors.

## INTRODUCTION

Breast cancer is the most common cancer and a major cause of death among women worldwide^[^^1 ▶^^]^. Thousands of new cases of breast cancer are diagnosed annually in Iran, which imposes a major burden on the health sector^[^^2 ▶^^,^^3 ▶^^]^. Breast cancer is classified into three main subsets of gene expression: luminal (A and B), basal-like, and Her2/neu-overexpressing^[^^4 ▶^^]^. TNBC is characterized by the absence of the expression of Her2, PRs, and ERs^[^^4 ▶^^]^. TNBC is a very aggressive subtype with a poor prognosis compared to other types of breast cancer, but accounts for less than 20% of cases. It has been shown that patients with TNBC have larger tumors, and a higher grade and invasion of the lymph nodes^[^^5 ▶^^]^. Studies have also displayed that only 20%-30% of the patients respond to common treatments such as chemotherapy and radiotherapy. In the remaining cases, there is recurrence of disease and resistance to treatment within the first three years after diagnosis^[^^6 ▶^^,^^7 ▶^^]^. A major problem related to breast cancer is late detection, especially in TNBCs, which are often diagnosed at advanced stages with a high histological grade^[^^8 ▶^^,^^9 ▶^^]^. Another issue is the lack of reliable early diagnostic biomarkers for TNBC. Therefore, documentation of early-detection biomarkers and the introduction of novel therapies to enhance the pathologicl complete response rate in TNBC patients are of great importance and need to be considered seriously.

Epigenetic variations have been demonstrated to play a role in the progress of breast cancer. These changes are involved in the expression of genes and, thus, disrupt the signaling pathways^[^^10 ▶^^]^. One of the most important mechanisms of epigenetics that causes cancer is histone change. The changes in histone structure due to environmental variations in different geographical regions and ethnicities can be dissimilar. Researchers are currently studying HDAC and its inhibitors. Recent investigations have displayed a direct correlation between breast tumors and differences in histone proteins^[^^11 ▶^^,^^12 ▶^^]^.

Equilibrium between the acetylation and deacetylation of nuclear histones is essential for the regulation of gene expression. These reactions are induced by HDAC and histone acetyltransferase, respectively. Aberrant expression of HDAC, also known as lysine deacetylase, has been linked to tumor progression, prognosis, and pathogenesis^[^^13 ▶^^,^^14 ▶^^]^. Class I HDACs are expressed in most tissues and include HDAC 1, 2, 3, and 8. The aberrant expression of class I HDAC has been shown in gastric, esophageal, colorectal, prostate, breast, and lung cancers^[^^15 ▶^^-^^17 ▶^^]^. HDAC8 is a recently recognized subtype of class I HDAC, which is associated with several malignancies. HDAC8 inhibition in T-cell lymphoma increases apoptosis in these cells, and its overexpression plays a significant role in the pathogenesis of neuro-blastoma^[^^18 ▶^^]^. In recent years, attention has been focused on the targeted inhibition of HDAC8 to control and inhibit the cancer growth^[^^19 ▶^^]^.

Despite the well-recognized roles of HDAC8 in the progression of breast cancer, its diagnostic value has not been considered. The current study was conducted to evaluate the possible association between HDAC8 expression and clinical outcomes of patients with breast cancer. The relation of this gene with the pathologic findings of TNBC patients were compared with notes of nTNBC subjects. The diagnostic value of HDAC8 in differentiation between TNBC and nTNBC subjects was also assessed.

## MATERIALS AND METHODS

**Patients**

Cancerous and adjacent normal tissue specimens were obtained from 50 patients with breast cancer who underwent surgery at Towhid Hospital in the city of Sanandaj in Iran between January 2016 and October 2018. The patients with breast cancer had an average age of 48.33 ± 9.5 years. None of the patients enrolled in this study had undergone pre-operative chemotherapy, radiotherapy, or any other treatment. Those with a history of tumors in other organs were excluded. The tumor, node, metastasis staging system and the Scarf-Bloom-Richardson criteria were used for staging and grading, respectively^[^^20 ▶^^,^^21 ▶^^]^. All specimens were immediately frozen in liquid nitrogen and stored at -80 °C pending assay. Detailed clinical information of the enrolled patients is listed in Table 1.

**Immunohistochemistry** **assay**

Tissues were excised, and immunohistochemistry was carried out. The tissues were then examined for PR, Her2, and ER. Staining was performed according to the standard protocol^[^^22 ▶^^]^.

**RNA purification, cDNA synthesis, and qRT-PCR**

RNX-Plus kits (Cinnagen, Iran) were used to extract the total RNA from the breast cancer specimens and adjacent normal tissue, according to the instructions provided by the manufacturer. The quality and quantity of the samples were assessed photometrically and electrophoretically. A RevertAid First Strand cDNA Synthesis Kit (Fermenta, Thermo Scientific, USA) was used to synthesize the cDNA from the total RNA, as per manufacturer’s instructions. Rotor-Gene 6000 (Corbett Research, Australia) was employed to perform qRT-PCR in triplicate in a 25-μL reaction volume consisting of 2× Maxima SYBR Green qPCR Master Mix buffer (Fermentas), 400 nM each of forward and reverse primer, and cDNA. Then 40 cycles of PCR were carried out as follows: 95 °C for 10 min for one cycle, 94 °C for 10 s, and 60 °C for 40 s. The expression of HPRT was utilized to normalize data from each group. The following primer pairs were also used for qPCR reaction: HDAC8 (FP) GGCTGCGGA ACGGTTTTAAG, HDAC8 (RP) GCTTCAATCAAA GAATGCACCATAC, HPRT (FP) CCTGGCGTCGT GATTAGTG, and HPRT (RP) TCAGTCCTGTCCAT AATTAGCC. The Ct values for each sample were determined with amplification plots in the logarithmic phase. The PCR outcome and efficiency of amplification were determined using LinRegPCR software V12.17 using the 2^−ΔΔCt^ method^[^^23 ▶^^,^^24 ▶^^]^.

**Table 1:** Comparison of clinicopathologic characteristics with age and tumor site in TNBC and nTNBC

**Clinical features**	**TNBC**		**nTNBC**	
	**n = 23 (%)**		**n = 27 (%)**	
Age of diagnosis				
<46	10	43.5	9	33
>46	13	56.5	18	67
				
Site of tumor				
Left	13	56.5	15	55.5
Right	10	43.5	12	44.5
				
ER				
Negative	23	100	10	37
Positive	0	0	17	63
				
PR				
Negative	23	100	14	50
Positive	0	0	14	50
				
Her2 status				
Negative	23	100	16	59.2
Positive	0	0	11	40.8
				
Tumor size				
≤3	12	52	14	52
>3	11	48	13	48
				
Lymphatic invasion				
Yes	11	52	14	52
No	12	48	13	48
				
Perineural invasion				
Yes	9	39	8	29.5
No	14	61	19	70.5
				
Grade				
Grade I + II	11	48	16	59.2
Grade III	12	52	11	40.8
				
Necrosis				
Yes	15	65.2	10	37
No	8	34.8	17	63
				
Histological type				
Ductal	16	69.5	18	66.6
Lobular	5	21.7	7	25.9
Other	1	4.3	2	7.4
Unknown	1	4.3	0	-

**Statistical analysis**

GraphPad Prism 7 was used for the statistical analysis. All data were expressed as mean ± standard error of three independent experiments, and *p* < 0.05 was considered statistically significant. The ROC curve was constructed to establish a sensitivity-specificity relationship. Cut-off values that provided the best sensitivities and specificities were determined. The sensitivity, specificity, positive and negative predictive values, positive and negative likelihood ratios, and accuracy were calculated^[^^25 ▶^^]^.

**Fig. 1:**
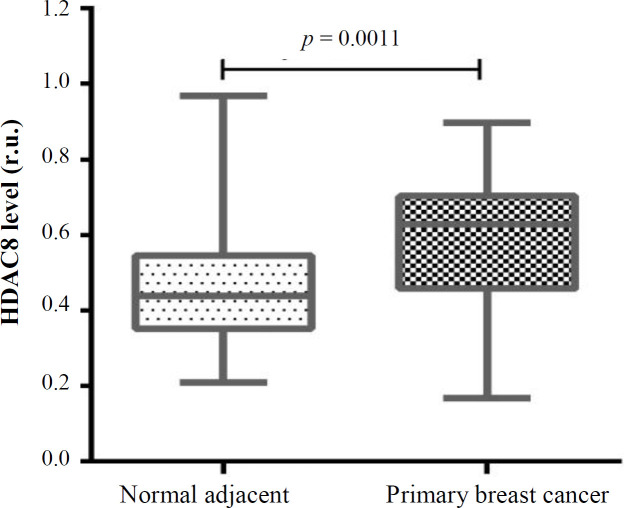
Expression of HDAC8 in cancerous and benign tissues

**Ethical statement**

The above-mentioned sampling protocols were approved by the Regional Ethics Committee of Kurdistan University of Medical Sciences, Sanandaj, Iran (ethical code: IR.MUK.REC.1395/279). Written informed consents were obtained from all the participants before surgery.

## RESULTS

**Fig. 2.**
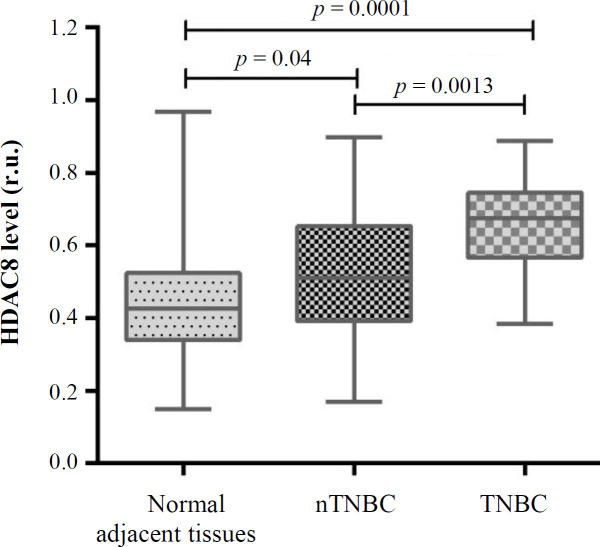
Expression of HDAC8 in normal specimens, nTNBC and TNBC

Fifty patients with breast cancer were included in the present study. Of these, 27 were nTNBC and 23 were TNBC. The expression level of HDAC8 increased significantly in the breast cancer samples compared to the normal tissue samples (0.5867 ± 0.023 vs. 0.4724 ± 0.024 [ru], respectively; *p* = 0.0011; Fig. 1). The results of the HDAC8 expression in the TNBC and nTNBC groups revealed that HDAC8 gene expression in both groups altered significantly in comparison to the benign tissue (*p* < 0.0001 and *p* = 0.04, respectively; Fig. 2). Also, a significant elevation was observed in the expression of HDAC8 gene in TNBC compared to the nTNBC patients (0.6694 ± 0.02 vs. 0.5162 ± 0.03 [ru], respectively; *p* = 0.0013; Fig. 2). The data showed that the overexpression of HDAC8 is a potential risk factor for the progression of TNBC (odds ratio = 6.729; 95% CI = 1.939-23.356; *p* = 0.002).

**Fig. 3:**
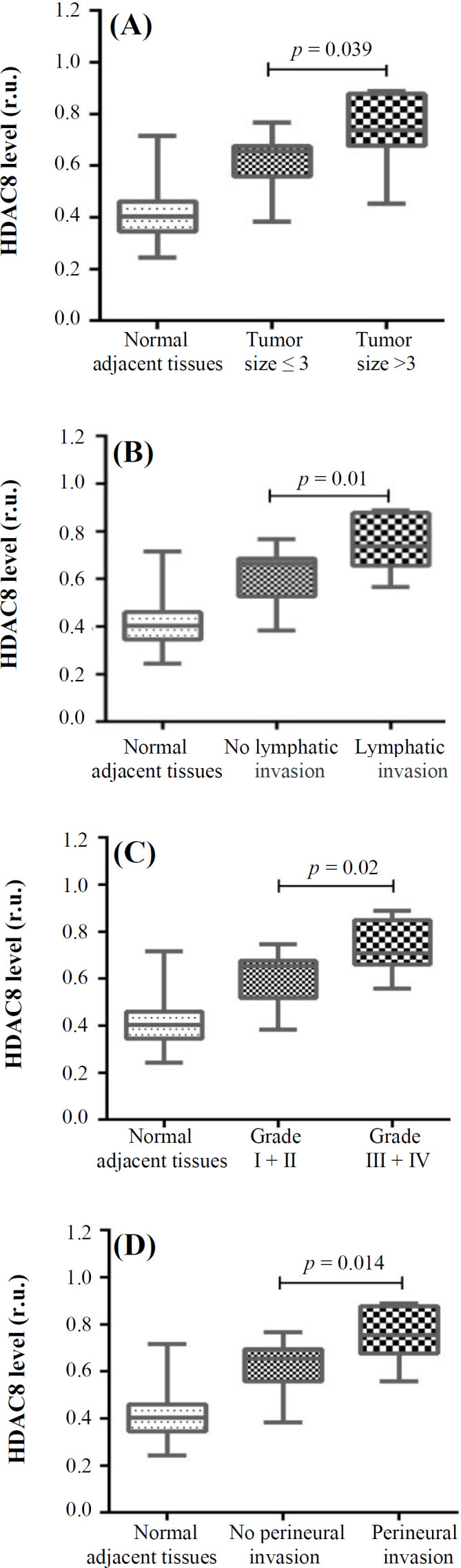
Association between the overexpression of HDAC8 with clinical findings in TNBC subjects. The Figure shows the relation between HDAC8 expressions (r.u.) with (A) tumor size (cm), (B) lymphatic invasion, (C) tumor grade, and (D) perineural invasion in TNBC subjects

The association between HDAC8 expression and pathological outcomes was studied in the nTNBC and TNBC groups. In nTNBC patients, there was no significant relationship between HDAC8 expression and tumor characteristics, including tumor size (*p *= 0.1), lymphatic invasion (*p* = 0.06), tumor grade (*p* = 0.14), and perineural invasion (*p* = 0.2). However, in the TNBC group, a significant association was found between the increased HDAC8 expression and tumor characteristics, including tumor size (*p* = 0.039; Fig. 3A), lymphatic invasion (*p* = 0.01, Fig. 3B), tumor grade (*p* = 0.02; Fig. 3C), and perineural invasion ( (*p* = 0.014; Fig. 3D). 

**Fig. 4:**
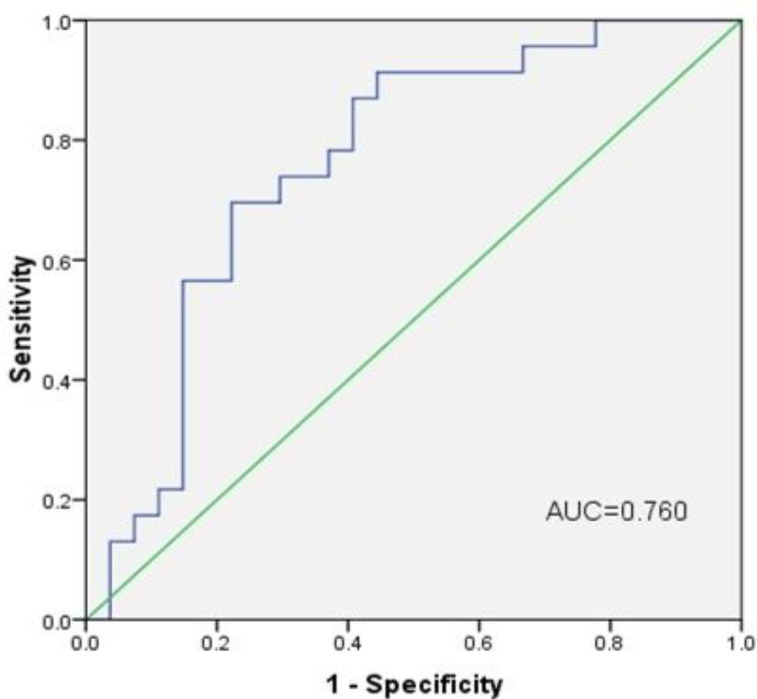
ROC curve for HDAC8

The diagnostic value of HDAC8, as a potential tumor marker, for the differentiation of nTNBC from TNBC subjects was investigated. The ROC curve was plotted, and the cut-off value was determined at 0.6279 (ru). Using that cut-off point, the AUC was 0.760 (95% CI = 0.624-0.896; Fig. 4). According to the cut-off point, the diagnostic value was determined as follows: sensitivity (73.91%), specificity (70.37%), positive predictive value (0.68), negative predictive value (0.76), positive likelihood ratio (2.49), negative likelihood ratio (0.37), and accuracy (72%). 

## DISCUSSION

Studies have revealed an oncogenic role for HDAC8 in the progression of breast cancer and have indicated the effect of this gene on TNBC. It has been shown that the *HDAC8* gene can regulate the biological function of cancer cells both in the cytoplasm and cell nucleus^[^^26 ▶^^,^^27 ▶^^]^. Overexpression of HDAC8 has been found to increase the migration of breast cancer cells through the Hippo signaling pathway. Consequently, suppression of the HDAC8 gene has been proposed as a potential target for TNBC treatment^[^^28 ▶^^]^. WU *et al.*^[^^29 ▶^^]^ have disclosed that suberoylanilide hydroxamic acid inhibits HDAC8/FOXA1 signals in the epithelial-mesenchymal transition of cancer cells and introduced this acid as an anti-tumor agent for the treatment of TNBC cancer. HDAC8 also is involved in cellular invasion and expression of the MMP-9 gene in human cancer cells^[^^30 ▶^^]^. Hypomethylation of the HDAC8 promoter significantly enhances the expression of this oncogene in TNBC cells^[^^31 ▶^^]^. Overexpression of HDAC8 has been indicated to be associated with poor prognosis and drug resistance in breast cancer^[^^31 ▶^^]^. It has also been shown that the HDAC8 gene is involved in regulating P53 expression and suppressing HDAC8/YY1 signals that may reverse the tumorigenicity of mutant P53 in breast cancer^[^^32 ▶^^]^. In line with previous studies, the current findings suggested a significant elevation of HDAC8 in breast cancer tissue specimens compared to normal adjacent tissues samples. It was also found the expression of this gene in TNBC cancer is much higher than in nTNBC. More interestingly, there was a direct significant association with clinical outcomes in TNBC subjects.

Despite the tumorigenicity of HDAC8, the value of this marker for use as a diagnostic tool has not yet been determined. On the other hand, the late diagnosis of TNBC increases the complexity of treatment, providing a poor prognosis for this type of cancer. Hence, it is necessary to develop new methods and novel tumor markers for early detection of TNBC. 

To the best of our knowledge, this study, for the first time, assesses the diagnostic utility of HDAC8, as a potential tumor marker for differentiation of TNBC from nTNBC subjects. To date, no other published studies have been found on the value of HDAC8 in TNBC patients. The current study used a cut-off point of 0.6279 (ru) and sensitivity and specificity of 73.91% and 70.37%, for HDAC8, respectively.

The present study highlights the up-regulation of HDAC8 from tissue as a diagnostic and prognostic 

marker. It was possible to validate the overexpression of HDAC8 in breast cancer specimens; therefore, it was determined that HDAC8 is higher in TNBC samples than in nTNBC and benign tissue. A correlation between tissue levels of HDAC8 and clinical findings was observed in the TNBC patients in this study.

In conclusion, our data indicate the diagnostic value of HDAC8 in breast cancer patients and the results suggest that it could serve as a predictor of breast cancer development.

## References

[B1] Benson JR, Jatoi I (2012). The global breast cancer burden. Future oncology.

[B2] Otaghvar HA, Hosseini M, Tizmaghz A, Shabestanipour G, Noori H (2015). A review on metastatic breast cancer in iran. Asian pacific journal of tropical biomedicine.

[B3] Farhood B, Geraily G, Alizadeh A (2018). Incidence and mortality of various cancers in iran and compare to other countries: A review article. Iran journal public health.

[B4] Perou CM, Sorlie T, Eisen MB, van de Rijn M, Jeffrey SS, Rees CA, Pollack JR, Ross DT, Johnsen H, Akslen LA, Fluge O, Pergamenschikov A, Williams C, Zhu SX, Lonning PE, Borresen-Dale AL, Brown PO, Botstein D (2000). Molecular portraits of human breast tumours. Nature.

[B5] Malla RR, Kumari S, Gavara MM, Badana AK, Gugalavath S, Kumar DKG, Rokkam P (2019). A perspective on the diagnostics, prognostics, and therapeutics of microRNAs of triple-negative breast cancer. Biophysical reviews.

[B6] Yin WJ, Lu JS, Di GH, Lin YP, Zhou LH, Liu GY, Wu J, Shen KW, Han QX, Shen ZZ, Shao ZM (2009). Clinicopathological features of the triple-negative tumors in chinese breast cancer patients. Breast cancer research and treatment.

[B7] Valentin MD, da Silva SD, Privat M, Alaoui-Jamali M, Bignon YJ (2012). Molecular insights on basal-like breast cancer. Breast cancer research and treatment.

[B8] Denkert C, Liedtke C, Tutt A, von Minckwitz G (2017). Molecular alterations in triple-negative breast cancer-the road to new treatment strategies. Lancet.

[B9] Jhan JR, Andrechek ER (2017). Triple-negative breast cancer and the potential for targeted therapy. Pharmacogenomics.

[B10] Bhat SA, Majid S, Wani HA, Rashid S (2019). Diagnostic utility of epigenetics in breast cancer-A review. Cancer treatment and research communications.

[B11] DeVaux RS, Herschkowitz JI (2018). Beyond DNA: The role of epigenetics in the premalignant progression of breast cancer. Journal mammary gland biology neoplasia.

[B12] Temian DC, Pop LA, Irimie AI, Berindan-Neagoe I (2018). The epigenetics of triple-negative and basal-like breast cancer: current knowledge. Journal breast cancer.

[B13] Ediriweera MK, Tennekoon KH, Samarakoon SR (2019). Emerging role of histone deacetylase inhibitors as anti-breast-cancer agents. Drug discovery today.

[B14] Zucchetti B, Shimada AK, Katz A, Curigliano G (2019). The role of histone deacetylase inhibitors in metastatic breast cancer. Breast.

[B15] Cao LL, Song X, Pei L, Liu L, Wang H, Jia M (2017). Histone deacetylase HDAC1 expression correlates with the progression and prognosis of lung cancer: A meta-analysis. Medicine (Baltimore).

[B16] Qiao W, Liu H, Liu R, Liu Q, Zhang T, Guo W, Li P, Deng M (2018). Prognostic and clinical significance of histone deacetylase 1 expression in breast cancer: A meta-analysis. Clinica chimica acta.

[B17] Mishra VK, Wegwitz F, Kosinsky RL, Sen M, Baumgartner R, Wulff T, Siveke JT, Schildhaus HU, Najafova Z, Kari V, Kohlhof H, Hessmann E, Johnsen SA (2017). Histone deacetylase class-I inhibition promotes epithelial gene expression in pancreatic cancer cells in a BRD4- and MYC-dependent manner. Nucleic acids research.

[B18] Heimburg T, Kolbinger FR, Zeyen P, Ghazy E, Herp D, Schmidtkunz K, Melesina J, Shaik TB, Erdmann F, Schmidt M, Romier C, Robaa D, Witt O, Oehme I, Jung M, Sippl W (2017). Structure-based design and biological characterization of selective histone deacetylase 8 (HDAC8) inhibitors with anti-neuroblastoma activity. Journalmedicinal chemistry.

[B19] Amin SA, Adhikari N, Jha T (2018). Structure-activity relationships of HDAC8 inhibitors: non-hydroxamates as anticancer agents. Pharmacol research.

[B20] Elston C (2005). Classification and grading of invasive breast carcinoma. Verhandlungen der deutschen gesellschaft fur Pathologie.

[B21] Edge SB, Compton CC (2010). The american joint committee on cancer: the 7th edition of the AJCC cancer staging manual and the future of TNM. Annals of surgical oncology.

[B22] Zarei F, Menbari MN, Ghaderi B, Abdi M, Vahabzadeh Z (2017). Higher risk of progressing breast cancer in kurdish population associated to CDH1 -160 C/A polymorphism. EXCLI journal.

[B23] Ruijter JM, Ramakers C, Hoogaars WM, Karlen Y, Bakker O, van den Hoff MJ, Moorman AF (2009). Amplification efficiency: linking baseline and bias in the analysis of quantitative PCR data. Nucleic acids research.

[B24] Tuomi JM, Voorbraak F, Jones DL, Ruijter JM (2010). Bias in the Cq value observed with hydrolysis probe based quantitative PCR can be corrected with the estimated PCR efficiency value. Methods.

[B25] Nikkhoo B, Sigari N, Ghaderi B, Afkhamzadeh A, Azadi NA, Mohsenpour B, Fathi F, Abdi M (2013). Diagnostic utility of adenosine deaminase in serum and bronchoalveolar lavage fluid for screening lung cancer in western iran. Journal of medical biochemistry.

[B26] Guo X, Ruan H, Li X, Qin L, Tao Y, Qi X, Gao J, Gan L, Duan S, Shen W (2015). Subcellular localization of class I histone deacetylases in the developing xenopus tectum. Front cell neurosci.

[B27] Ha SD, Han CY, Reid C, Kim SO (2014). HDAC8-mediated epigenetic reprogramming plays a key role in resistance to anthrax lethal toxin-induced pyroptosis in macrophages. Journal immunol.

[B28] An P, Li J, Lu L, Wu Y, Ling Y, Du J, Chen Z, Wang H (2019). Histone deacetylase 8 triggers the migration of triple negative breast cancer cells via regulation of YAP signals. European journal of pharmacology.

[B29] Wu S, Luo Z, Yu PJ, Xie H, He YW (2016). Suberoylanilide hydroxamic acid (SAHA) promotes the epithelial mesenchymal transition of triple negative breast cancer cells via HDAC8/FOXA1 signals. Biological chemistry.

[B30] Park SY, Jun JA, Jeong KJ, Heo HJ, Sohn JS, Lee HY, Park CG, Kang J (2011). Histone deacetylases 1, 6 and 8 are critical for invasion in breast cancer. Oncology reports.

[B31] Hsieh CL, Ma HP, Su CM, Chang YJ, Hung WY, Ho YS, Huang WJ, Lin RK (2016). Alterations in histone deacetylase 8 lead to cell migration and poor prognosis in breast cancer. Life sciences.

[B32] Wang ZT, Chen ZJ, Jiang GM, Wu YM, Liu T, Yi YM, Zeng J, Du J, Wang HS (2016). Histone deacetylase inhibitors suppress mutant p53 transcription via HDAC8/YY1 signals in triple negative breast cancer cells. Cell signal.

